# Vertical bone augmentation with titanium granule blocks in rabbit calvaria

**DOI:** 10.1002/cre2.67

**Published:** 2017-07-03

**Authors:** Peter Abrahamsson, Dan‐Åke Wälivaara, Jonas Anderud, Ryo Jimbo

**Affiliations:** ^1^ Hallands Hospital Maxillofacial Unit SE Sweden; ^2^ Department of Oral and Maxillofacial Surgery and Oral Medicine Malmö University SE Sweden

**Keywords:** animals, bone regeneration, bone substitutes, rabbits

## Abstract

To determine whether it is possible to vertically augment bone utilizing a block graft from compressed titanium granules mainly used previously for contained bone defects and to determine whether there exists a difference in osteoconductive properties between the white and the grey granules. In 11 rabbits, 4 titanium blocks were inserted on each rabbit's skull bone according to a randomized scheme. These blocks were made from standardized compressed titanium granules. Type A: PTG grey, small granules (Pourus Titanium Granules, Tigran, Malmö, Sweden); Type B: PTG grey, large granules; Type C: PTG white, small granules; Type D: PTG white large granules. After 12 weeks, the animals were sacrificed and specimens were collected for histology and μCT scanning. From both the μCT and histology, it can be said that bone formation was successfully achieved for all groups, and the granules maintained their volume. The histomorphometric BA (bone area) evaluation in the entire grafted area presented that there were no statistical differences between all groups tested. The lowest 1/4 BA in contact with the rabbit skull presented that groups A and C presented the highest mean BA, and group A presented significantly higher BA than that of group D (*p* = 0,049). No significant differences were noted between groups A, B and C. Within the limitation of this study, no differences were noted between small white or grey PTG blocks. The large granules presented less bone ingrowth area compared to the small granules and this trend was regardless of the different PTG types. The entire grafted area was not filled with new bone suggesting that bone migration occurred mostly from the existing cortical bone side suggesting contact osteogenesis.

1

After tooth extraction, the alveolar bone undergoes remodeling, which results in vertical and horizontal bone resorption (Araujo & Lindhe, 2005). This has been suggested to be an inevitable phenomenon because the so‐called bundle bone is nourished by the microvessels extended from the periodontal ligament (Araujo & Lindhe, 2005). Thus, subsequently after the loss of the periodontal ligament due to tooth extraction, it is of natural course that vertical bone resorption takes place. In cases with severe bone resorption, reconstruction of the alveolar process is often necessary prior to the placement of dental implants.

The gold standard for bone augmentation is to harvest autogenous bone and is commonly used in oral maxillofacial reconstructive surgery. However, the autogenous bone is known for its rapid resorption during the healing phase, and some reports indicate that approximately 40% of the graft will resorb during the early phase (Abrahamsson, Walivaara, Isaksson, & Andersson, 2012; Cordaro, Amade, & Cordaro, 2002). As a result, this could lead to the collapse of the augmented site, which may affect the subsequent implant placement. Another concern with the autogenous bone grafting is the invasive properties of the procedure, which at times generate postoperative complications (Yamada et al., 2013). Thus, as an alternative, the use of biomaterials has obtained some popularity in cases where harvesting of bone grafts are restricted. However, some concerns do remain with regard to the use of some biomaterials. Especially the use of xenografts or allografts has been of specific concern because the risk of unknown disease transmission cannot fully be denied (Tovar et al., 2014). A possible candidate for an off the shelf bone substitute is the titanium oxide particles. Because titanium is a well‐documented material used in dental implants, it was of interest to test the osteogeneic properties of these materials as bone substitutes. The so‐called porous titanium granules (PTGs) have in a rabbit model showed total bone fill in the porosities of the granules 16 weeks after surgical insertion in rabbit tibia (Rubshtein, et al. 2014). PTGs have also been applied clinically, and reports indicate comparable results when mainly applied in the sinus (Dursun et al., 2015; Lambert et al., 2013; Vandeweghe, Leconte, Ono, Coelho, & Jimbo, 2013) or in socket preservations (Tavakoli et al., 2012; Verket, Lyngstadaas, Ronold, & Wohlfahrt, 2014) and in reconstruction of peri‐implant osseus defects (Jepsen et al., 2016). PTG has been used in critical size defects on rabbit tibia. The PTG particles showed osteoconductive properties, but the authors concluded that the granules should be covered by a membrane in larger defects (Delgado‐Ruiz et al., 2014). However, the metallic color of the material restricted their clinical usage to contained sites, and it was difficult to be applied in esthetically demanding sites. In order to overcome these drawbacks, a further modification was conducted to improve the color of the titanium particles to white. The white porous titanium granules (WPTG) possess its color due to the modifications in the oxidation properties, which resembles the color of the commercially available calcium phosphate biomaterials. In a study on minipigs, 30 titanium implants were installed in bone defects, 11 weeks after reconstruction with PTG, WPTG, or a sham group. The study presented that there were no differences in osseointegration properties, regarding histomrorphometic bone to implantat contact 37% for WPTG, 67% for PTG, and 61% for the sham group, for implants placed after bone reconstruction (Verket et al., 2014), which is an indication that the modified material may be used with comparable prognosis before implant treatment as the original titanium granule.

A challenge for these materials is augmentation of the alveolar bone vertically or horizontally, which at times exceeds the biological envelope. Especially in the esthetic region, this is an important aspect because the soft tissue volume is in direct relation to the volume of the supporting bone. Clinicians might be tempted to conduct an overcorrection in order to obtain sufficient volume of the peri‐implant tissue.

The objectives of this study were to determine whether it is possible to vertically augment bone with a block graft from compressed titanium granules mainly used previously for contained bone defects and to determine whether there exists a difference in osteoconductive properties between the white and the grey granules with the different sizes of the granules.

## 2 MATERIALS AND METHODS

### 2.1 Materials

The study was approved by the Malmö/Lund regional animal ethics committee (approval no. M 378‐12). Four prefabricated titanium blocks were used in this project. They were made from standardized compressed titanium granules. Type A: PTG grey, small Granules (Porous Titanium Granules, Tigran, Malmö, Sweden), Type B: PTG grey, big granules, Type C: PTG white, small granules, Type D: PTG white large granules. The small granules have the size between 0.7 and 1.0 mm, and with a porosity of approximately 80%. The larger granules were between 2.0 and 3.0 mm. They were compressed to form test body with a diameter of 12 mm at the base and 7 mm at the top. The distance from the base to the highest point was 6 mm. They were compressed only to fit and have a stable form without braking on fixation to the bone. This was made to avoid breaking the structure of the individual granules. In the middle of the block, there was a prefabricated hole 2 mm in diameter. This hole was made to fit a titanium screw (De Puy Synthes, Oberdorf, Switzerland 04.503.203 Ø1.5 mm 8 mm self‐drilling).

### 2.2 Animals and anesthesia and surgery

**Figure 1:**
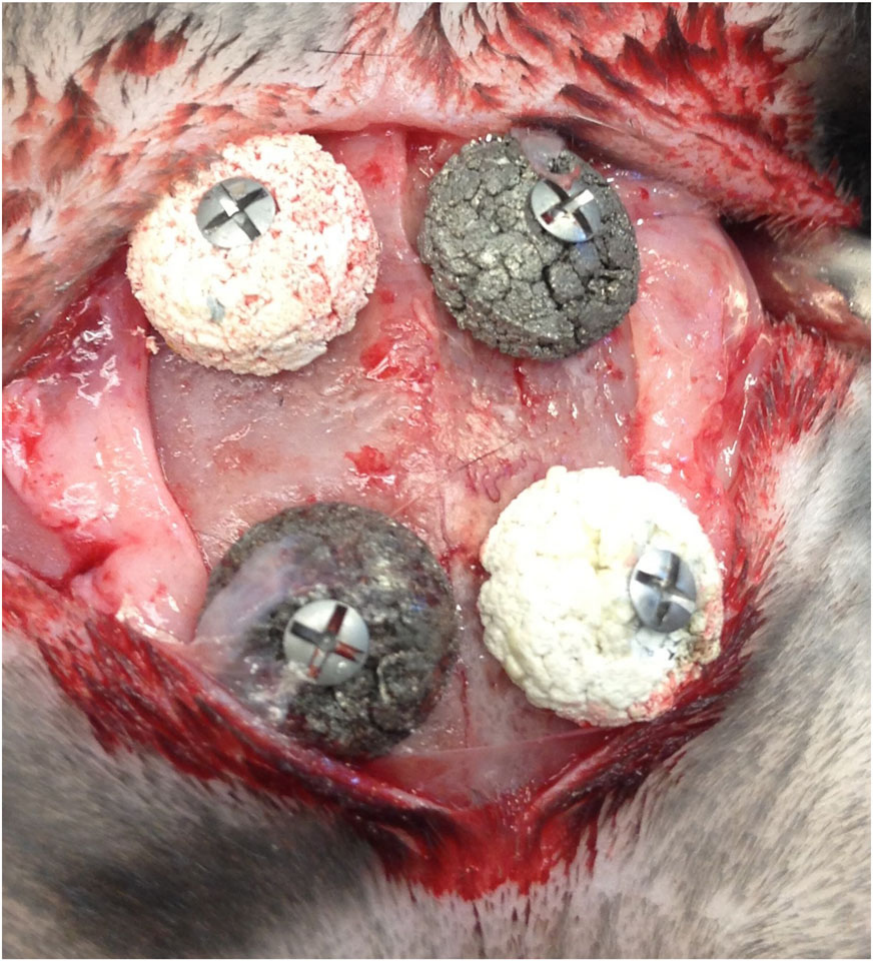
Four porous titanium granule blocks fixated to the rabbit skull titanium mini screws

Eleven lop‐eared rabbits of mixed sexes with a mean body weight of 4.29 kg were used in this study. Four implants were inserted on each rabbit's skull bone according to a randomized scheme. Preoperatively, the rabbit skulls were shaved and disinfected with chlorhexidine (5 mg/ml, Pharmacia AB, Stockholm, Sweden). The animals were anaesthetized by intramuscular injection with a mixture of 0.15 ml/kg medetomidine (1 mg/ml Dormitor; Orion Pharma, Sollentuna, Sweden) and 0.35 ml/kg ketamine hydrochloride (50 mg/ml Ketalar; Pfizer AB, Sollentuna, Sweden). At each insertion site 0.5 ml Lidocaine hydrochloride 2% (Xylocaine 10 mg/ml; AstraZeneca AB, Södertälje, Sweden) was used as local anesthetics. Sterile conditions were maintained during surgical procedures. A 3‐cm long incision through the skin and the periosteum was made along the central line on top of the skull. The periosteum was dissected from the bone. Four space titanium blocks were fixed to the skull with 1 titanium screws each (Figure 1). The blocks were covered by periosteum that was closed in position using Vicryl 4‐0 (Ethicon, 2000, Nordstedt, Germany). The skin was closed with continuous stitching. Postoperatively, buprenorphine hydrochloride (0.5 ml Temgesic; Reckitt Benckiser, Slough, UK) was administered as an analgesic for 3 days. No antibiotics were used. The rabbits were kept in single cages.

### 2.3 Animal sacrifice and preparation of samples

The rabbits were sacrificed with an overdose (60 mg/ml) of sodium pentobarbital (Apoteksbolaget AB, Stockholm, Sweden). Samples including the titanium blocks, including the attaching skull bone, and periosteum were retrieved and placed in 4% formaldehyde.

After fixation, the samples were gradually dehydrated in a series of ethanol solutions percentages ranging from 70 to 100. Following dehydration, the samples were embedded in methacrylate‐based resin (Technovit 7200 VLC; Heraeus Kulzer Wehrheim, Germany) according to the manufacturer's instructions.

The embedded samples were thereafter subjected to micro computed tomography (μCT) scanning for an overall observation of the bone formation. A detailed quantification could not be performed due to difficulties in removing the metallic halation of the particles.

In brief, a μCT equipment with a microfocus X‐ray tube (focus size 868 mm, MCT‐100MF; Hitachi Medical Corporation, Tokyo, Japan) was used to create a 3D image. The tube voltage, tube current, magnification, and voxel size were 70 kV, 100 μA, ×6, and 21.0 × 21.0 × 21.0 μm, respectively. The particles and the bony tissue were separated by 3D space filtration.

After μCT scanning, the samples were subjected to nondecalcified ground sectioning. In brief, the samples were cut by a diamond saw in the middle of the sample, and one central section was prepared to a final thickness of 30 μm. Thereafter, the sections were treated for 10 min with 10% H_2_O_2_ then stained in toluidine blue and pyronin G.

On these histologic sections, histological evaluations were performed using a light microscope (Eclipse ME600, Nikon, Tokyo, Japan) and histomorphometrical data were analyzed by an image analysis software (Image J ver.1.43u; National Institutes of Health, Bethesda, Maryland). The bone area (BA) within the graft material was calculated in two regions of interests.

1. BA within the entire graft material
2. BA within basal 1/4 (1,5 mm height of the basal area) of the entire graft material in contact to the original cortical bone bed.

### 2.4 Statistics

Statistical analysis was performed by one‐way analysis of variance and Tukey as post hoc was used for the statistical comparisons. Statistical significance was set at *p* = .05.

## 3 RESULTS

There were no complications during the healing period. All animals had gained weight (mean body weight = 4,45 kg during the healing period. All PTG blocks were stable at time of grafting, and there were no signs of infection.

**Figure 2:**
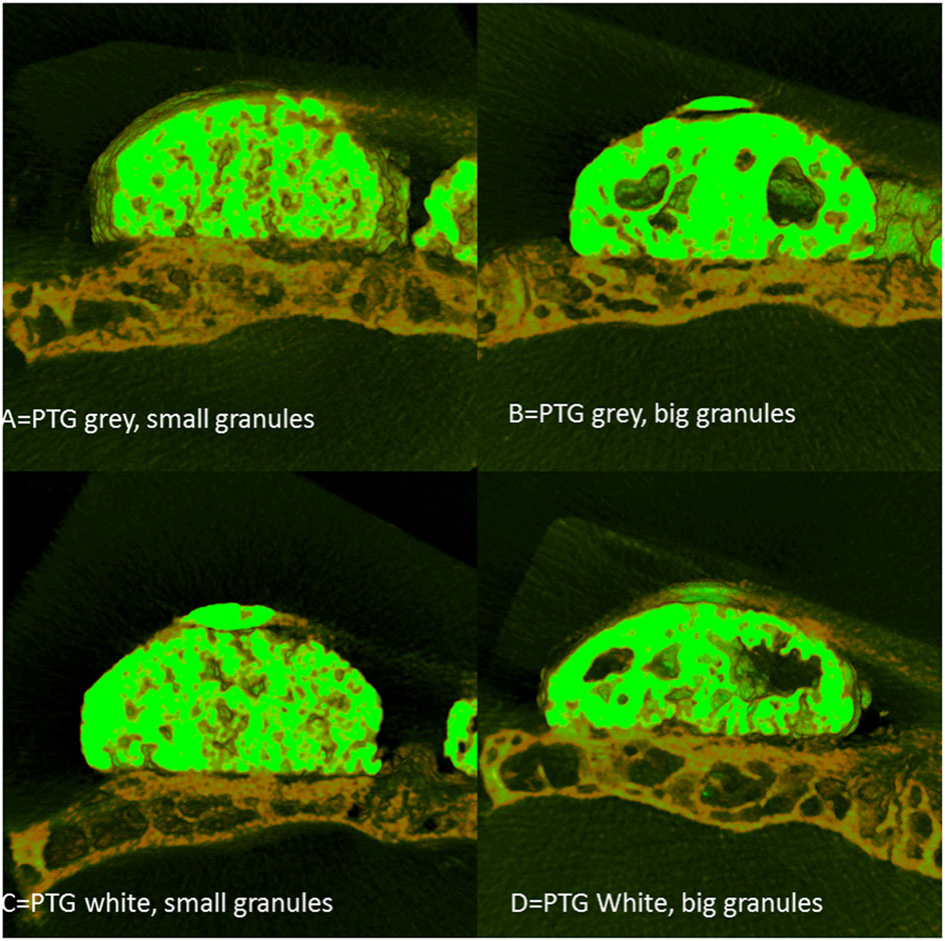
Three‐dimensionally reconstructed image of all groups tested. The color in light green represents the Ti granules, and the color in dark yellow represents bone

**Figure 3:**
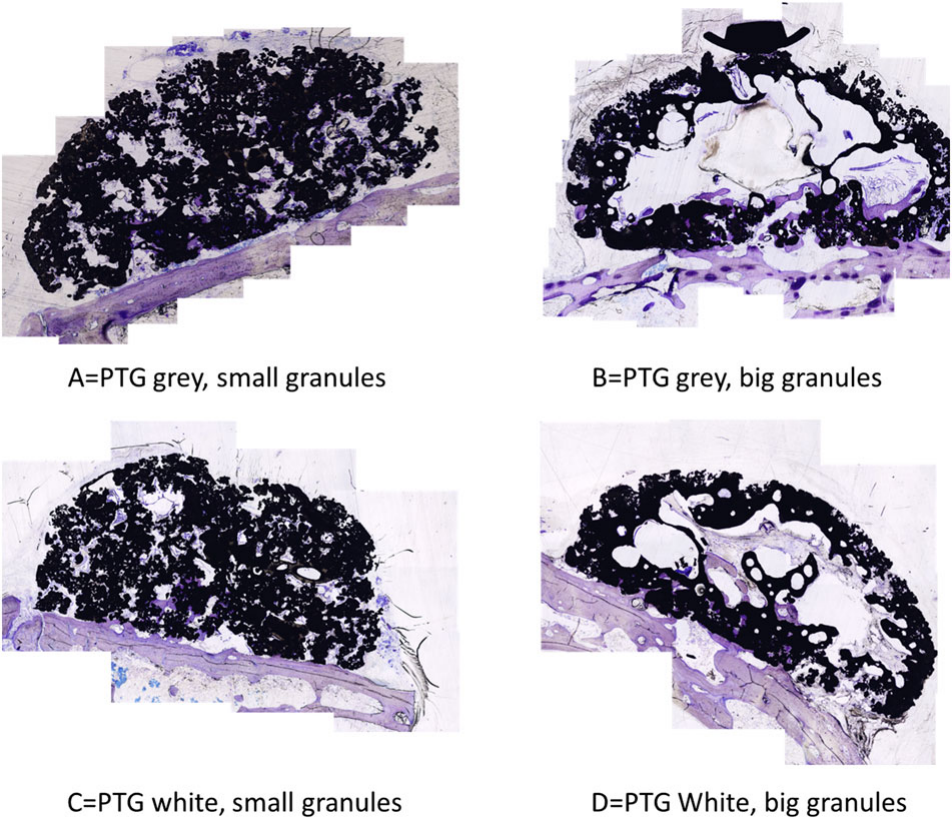
Histologic images of all groups tested. The big granules seemed to create a hollow structure. (original magnification ×10)

The morphological results of both μCT and histologic observation presented bone formation differences between the 4 groups (Figures 2 and 3). When considering the differences in granule sizes, it was evident that the groups with small granules were evenly distributed within the grafted volume. Whereas for the groups with large granules, there was a large defect in the center of the graft.

**Figure 4:**
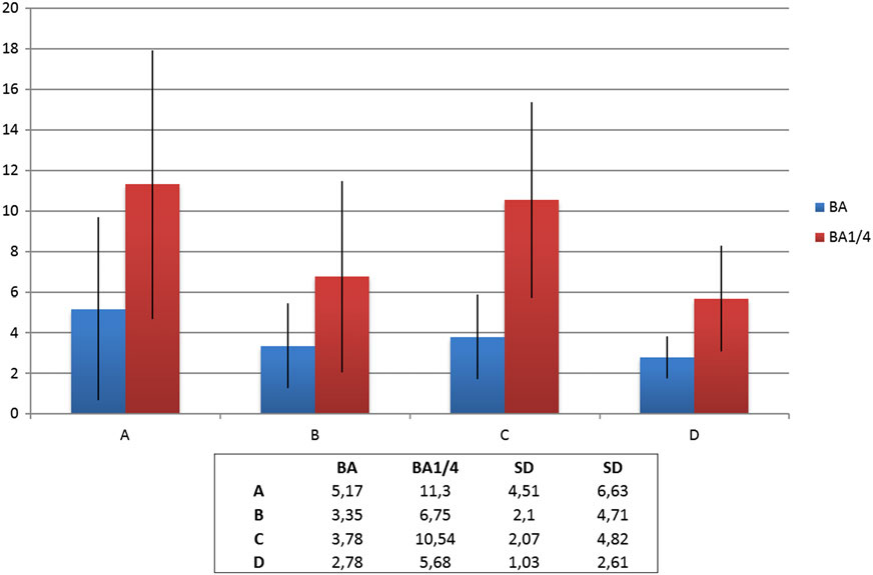
The 1/4 bone area (BA) % presented that groups A and C presented the highest mean BA, and group A presented significantly higher BA than that of group D (*p* = 0,049). No significant differences were noted between groups A, B and C

From both the μCT and histology, it was stated that bone formation was successfully achieved for all groups, and the granules maintained their volume. New bone was formed in direct contact with the PTG and the WTPG. Most of the bone was formed in the lowest ¼ of the blocks. The histomorphometric BA evaluation in the entire grafted area presented that there were no statistical differences between all groups tested (Figure 4). The 1/4 BA presented that groups A and C presented the highest mean BA, and group A presented significantly higher BA than that of group D (*p* = 0,049). No significant differences were noted between groups A, B and C (Figure 4).

Groups A and C had the highest mean BA in the lower areas although the differences were not significant.

## 4 DISCUSSION

The new bone in this study was more evenly distributed in the PTG groups with small granules, and the groups with larger granules presented large defects in the center of the graft. A similar situation has been presented when using 3D‐synthetic monetite blocks on the rabbit skull (Torres et al., 2011). Maximum bone levels were reached on the lateral walls and the lowest registration in the central region of the blocks. These blocks were cylindrical with a diameter of 9 mm and a height of 3 or 4 mm and fixed to the rabbit skull with a central titanium screw. The PTG blocks used in this study were 12 mm in diameter at the base and narrower at the top to mimic a dome trying to avoid sharp edges. The highest point was 6 mm from the base. Comparing this with the cylinders with a height of 4,5 mm used by Lundgren, Lundgren, Wennerberg, Hammerle, and Nyman (1999) and monetite blocks with a height of 3–4 mm used by Torres et al*.*, the height setup of this study was more challenging.

The histology and the micro‐CT showed that new bone ingrowth was noted within the region, where the PTG blocks were used. It was evident that there were more bone in the lower part of the PTG blocks and more soft tissue in the upper part. Most of the bone formation was formed in the lowest ¼ of the block representing the height of 1,5 mm. In accordance to Botticelli, Berglundh, and Lindhe (2004) premade 4 wall defects around dental implants in a dog model had normal bone healing in distances from 1 to 2.25 mm, furthermore when the buccal bone wall was intentionally removed the healing resulted in a defect resolution at the mesial, distal, and lingual aspects. At the buccal aspect, healing was incomplete due to limited bone formation from the lateral and apical parts of the defect (Botticelli, Berglundh, & Lindhe, 2004). This is in accordance to this study where there were no supporting bone walls and the bone formation only could form from the basal bone, resulting in bony defects in the upper part of the PTG blocks. The reason for this can be explained by the fact that no membrane was used to cover the PTG blocks leaving the upper part in direct contact to the soft tissue. The soft tissue could then grow into the upper part of the blocks before the bone had grown into this area. Delgado‐Ruiz et al. (2014) demonstrated in a critical size defect on rabbit tibia that a membrane was essential to control PTG particle migration, promote clot stabilization and to separate the PTG from undesired soft tissue. This was most important in larger defects (Delgado‐Ruiz et al., 2014), suggesting that a collagen membrane should have been used to cover the blocks in this study. Still, a collagen membrane might not be sufficient to protect the grafted area from ingrowing soft tissue. In a rabbit study when using deproteinized bovine bone mineral (DBBM) protected by a titanium mesh and a collagen membrane, the top part of the graft still had soft tissue between the DBBM particles as seen in the top part of the PTG‐blocks in this study (Abrahamsson, Isaksson, Gordh, & Andersson, 2010). Lundgren et al. (1999) used titanium cylinder as a barrier device to guide bone from the rabbit skull. New bone was formed in direct contact to the titanium walls. However, the full volume of newly created bone filled 77,9% ± 10,5% in blasted titanium and 73.4% ± in turned titanium, of the cylinder leaving an empty space close to the top of the cylinder (Lundgren et al., 1999).

In another rabbit study, Anderud et al (2014) used ceramic domes to guide bone outside the skeletal envelope on the rabbit skull. The 3D bone volumetric analysis found that more bone was created under a contained dome compared to a dome with open holes. The closed space facilitated bone growth without the soft tissue ingrowth or empty space in the top part of the dome. This suggests that more bone could have been formed in the whole of the PTG blocks if they were protected from the soft tissue with a nonresorbable occlusive membrane.

In sinus floor augmentation, PTG has shown oteoconductive properties (Bystedt & Rasmusson, 2009). The draw back in a clinical situation might be the metallic color from the titanium used. In this study, grey and white PTG was compared. No significant difference was noted in the formed BA between the small grey and white PTG. This is an indication that the white PTG can possibly be used in esthetically demanding areas such as the anterior maxilla instead of the grey colored PTG, which may result in discoloration of the gums.

All PTG blocks were embedded for μCT scan for overall three‐dimensional observation of the bone formation. Micro CT scan has been used to analyze bone formation in a bilateral sinus augmentation study comparing equine derived xenograft and xenograft mixed with PTG (Dursun et al., 2015). After 6 months of healing, bone biopsies were retrieved from 16 implant sites and processed for μCT scanning. No difference was found between the groups according to bone structural parameters.

The advantage with the PTG is that the material does not resorb therefore the risk for volumetric collapse is less significant. It is well known that when using autologous bone blocks, 18–28% of the graft will be resorbed (Abrahamsson et al., 2012; Cordaro, Torsello, Morcavallo, & di Torresanto, 2011; Cordaro et al., 2002; Maiorana, Beretta, Salina, & Santoro, 2005), and clinicians often use a mixture of less resorbing biomaterials to compensate for this draw back. For instance, DBBM and collagen barrier membrane has been added to the bone block graft in patients, which has resulted in less resorption (Maiorana et al., 2005). On the other hand, it could relate to a higher risk for complications such as postoperative infection (Cordaro et al., 2011). The results obtained from this study suggested that no complications or infections were noted; however, it must be stressed here that in the clinical situation, this is still something to consider if using titanium blocks to widen the alveolar crest in patients, in combination with dental implants.

Guiding bone outside the skeletal envelope is considered a challenge. The majority of the newly formed bone was formed in the lower 1/4 in contact to the original cortical bone bed. The same pattern can be seen in similar augmentations in humans using human allografts. Spin‐Neto et al*.* (2015) compared cortical (AL‐C), corticocancellous (AL‐CC) fresh‐frozen block bone allografts to cortical block bone autografts (AT) for lateral ridge augmentation. After 6–8 months of healing, biopsies were harvested at the same time implants were placed. AT bone blocks showed 25% vital bone, and the rest was necrotic bone and soft tissue. The AL‐CC presented 9% vital bone and the AL‐C only 3%. Benic et al. (2016) compared prepared defects in a dog mandible filled with DBBM particles, DBBM blocks, and equine blocks to empty controls. The empty control was less effective in re‐establishing the ridge contour compared to all other groups. Dental implants were installed at the time for augmentation, and the histologic finding after 4 months of healing presented considerably higher new bone formation at the lateral sections compared to the central sections of the graft. In the majority of the central sections, only a minor portion of the previously exposed implant surfaces were in contact with new bone. In all grafted groups, soft tissue represented more than 50% of the grafted area. These blocks were placed into premade defects; therefore, they had a larger direct bone contact from four surfaces of vital bone compared to the PTG blocks in this study that only had direct contact with one surface suggesting a longer distance for the new bone to grow to reach the top part of the block. In a clinical case report, a BBM (bovine bone mineral) block has successfully been used to augment an anterior alveolar defect. After 6 months of healing, a dental implant was installed and after 2 years of function, the implant was still functioning, and no changes in bone height were observed (Steigmann, 2008). Bone substitutes might be an interesting alternative to bone grafts, avoiding morbidity of the donor sites, and a nonresorbable graft material such as the PTG might be beneficial.

The strengths with this study are that the materials are tested on the same rabbit with no complications and the protocol is easy to randomize and standardize.

The draw backs of this study are that there were no sham group, no other materials tested that are well known, and no membranes were tested to cover the PTG blocks.

## 5 CONCLUSION

Bone can be augmented outside the skeletal envelope with a PTG block. Within the limitation of this study, no differences were noted between small white or grey PTG blocks. The large granules presented less bone ingrowth area compared to the small granules, and this trend was regardless of the different PTG types. PTG blocks with small granules presented a similar healing and new bone formation for both types (white or grey). The entire grafted area was not filled with new bone suggesting that bone migration occurred mostly from the existing cortical bone side suggesting contact osteogenesis. Further studies should be performed with white small particle PTG blocks with different dimensions to determine whether the height of the blocks influence the bone ingrowth. Furthermore, the stability of the newly formed bone under functional loading should be tested in order to determine whether osseointegration can be achieved in such regenerated bone sites.
